# Genetic effects on longitudinal cognitive decline during the early stages of Alzheimer’s disease

**DOI:** 10.1038/s41598-021-99310-z

**Published:** 2021-10-06

**Authors:** Atul Kumar, Maryam Shoai, Sebastian Palmqvist, Erik Stomrud, John Hardy, Niklas Mattsson-Carlgren, Oskar Hansson

**Affiliations:** 1 Clinical Memory Research Unit, Department of Clinical Sciences, Lund University, Malmö, Sweden; 2 UK Dementia Research Institute and Department of Neurodegenerative Disease and Reta Lila Weston Institute, UCL Queen Square Institute of Neurology and UCL Movement Disorders Centre, University College London, London, WC1V 6LJ UK; 3 Memory Clinic, Skåne University Hospital, Malmö, Sweden; 4 Institute for Advanced Study, The Hong Kong University of Science and Technology, Hong Kong SAR, China; 5 Department of Neurology, Skåne University Hospital, Lund, Sweden; 6 Wallenberg Centre for Molecular Medicine, Lund University, Lund, Sweden; 7 Faculty of Medicine, Biomedical Center, Lund University, Room No. C1112a, Sölvegatan 19, 22362 Lund, Sweden

**Keywords:** Computational biology and bioinformatics, Genetics, Neuroscience

## Abstract

Cognitive decline in early-stage Alzheimer’s disease (AD) may depend on genetic variability. In the Swedish BioFINDER study, we used polygenic scores (PGS) (for AD, intelligence, and educational attainment) to predict longitudinal cognitive change (measured by mini-mental state examination (MMSE) [primary outcome] and other cognitive tests) over a mean of 4.2 years. We included 260 β-amyloid (Aβ) negative cognitively unimpaired (CU) individuals, 121 Aβ-positive CU (preclinical AD), 50 Aβ-negative mild cognitive impairment (MCI) patients, and 127 Aβ-positive MCI patients (prodromal AD). Statistical significance was determined at Bonferroni corrected *p* value < 0.05. The PGS for intelligence (beta = 0.1, *p* = 2.9e−02) was protective against decline in MMSE in CU and MCI participants regardless of Aβ status. The polygenic risk score for AD (beta =  − 0.12, *p* = 9.4e−03) was correlated with the rate of change in MMSE and was partially mediated by Aβ-pathology (mediation effect 20%). There was no effect of education PGS on cognitive measures. Genetic variants associated with intelligence mitigate cognitive decline independent of Aβ-pathology, while effects of genetic variants associated with AD are partly mediated by Aβ-pathology.

## Introduction

The rate of cognitive decline in Alzheimer’s disease (AD) is highly variable^1,2^. A greater understanding of factors underlying this variability may provide tools for better prognostics and provide clues to novel treatments to modulate cognitive decline. Several potential factors may contribute to variability in cognitive decline, for example, the burden of AD pathology, cognitive reserve, concomitant pathologies, therapies and genetic variance^3^. Several genes contribute to the heritability of AD^4,5^, but studies testing genetic contributions to the rate of cognitive decline in AD are rare^6–8^. There is especially a lack of studies testing associations between genetic variants and cognitive decline during the early stages of AD, before dementia^9–11^. For example, it is unclear if genetic variants that are associated with AD diagnosis^12^, as well as with intelligence^13^ and education attainment^14^, may modulate cognitive decline during the earliest stages of AD. This is especially relevant given the increased focus on early disease stages (including preclinical AD) for potential disease-modifying treatments. Genetic variants that are associated with trajectories of cognitive decline during the early stages of the disease may be used to identify candidate treatment targets.

The relationship between genetic risk factors and cognitive decline can be tested simultaneously for individual and multiple genetic variants using the polygenic scoring (PGS) method^15^. For PGS, the cumulative effect of a large number of genetic variants across the entire genome is calculated by summing the weighted effect size of each variant on the target feature (e.g. AD) from a genome-wide association analysis (GWAS) multiplied by the number of effect alleles (0, 1, or 2) in that individual^16,17^. For a risk trait (e.g. AD), the term polygenic risk score (PRS) is used, while PGS is used for a benign or undecided trait (e.g. intelligence).

This study explored the relationship between known AD genetic risk factors and longitudinal cognitive impairment in the early stages of AD, including individuals with preclinical AD or mild cognitive impairment (MCI) due to AD. We hypothesized that genes associated with cognitive ability and/or genes associated with AD would impact changes in cognitive ability, and that these changes would be partly mediated by Aβ pathology. We used longitudinal cognitive data from the BioFINDER study and utilized three polygenic predictors: (1) a PRS for AD (PRS-Alz), (2) a PGS for intelligence (PGS-Int) and (3) a PGS for educational attainment (PGS-Edu) to ascertain the role of genetic variants that contribute to AD, intelligence and educational attainment as predictors of cognitive decline.

## Methods

### Study participants

The study population consisted of 381 cognitively unimpaired (CU) elderly participants and 177 patients with mild cognitive impairment (MCI) from the prospective and longitudinal Swedish BioFINDER sample (clinical trial no. NCT01208675; www.biofinder.se), for which baseline cognitive tests, age, education, gender, Aβ status and at least three time-points for MMSE (including baseline) were available. Details on recruitment, exclusion and inclusion criteria have been presented previously^18,19^ and detailed in the supplementary file. Among consecutively included patients with mild cognitive symptoms, some were classified as MCI and some as subjective cognitive decline (SCD). Following research guidelines, control participants and patients with SCDs were combined to make CU individuals^20^.

### Endophenotype

Longitudinal change in Mini-mental state examination (MMSE) score was used as the main outcome variable in this study. The MMSE is a commonly used brief cognitive test, which explores five cognitive function areas: orientation, registration, attention and calculation, word recall, and language, with a score ranging from 0 (worst) to 30^21^.

In addition, we also used four other longitudinal cognitive measures: ADAS-Cog word list delayed recall test (a test of episodic memory from Alzheimer’s Disease Assessment Scale)^22^, AQT (a Quick Test: A test of processing speed and executive function)^23^, TMT-B (Trail Making Test part B: a test of processing speed and executive function)^24^ and PACC (Preclinical Alzheimer Cognitive Composite: a composite sensitive to very early cognitive decline in AD)^24,25^.

### Genotyping and preparation of genetic data

Genotyping was conducted using the Illumina platform GSA-MDA v2. Subject-level and SNP level quality control (QC) was performed as per accepted guidelines^26^. In brief, person based QC included compatibility between chip-inferred gender and self-reported gender, call rates (1% cut-off), and extreme heterozygosity. In addition, high-quality variants (autosomal, bi-allelic variants with Hardy–Weinberg equilibrium (HWE) P > 5 × 10^−8^, Minor Allele Frequency [MAF] ≥ 5% [rather than, e.g. MAF > 1% due to the relatively small sample size of the cohort] and with a call rate of > 99%) were used. Further information on the imputation and QC process is detailed in supplementary methods.

### Polygenic score calculation

The PRS / PGS was calculated with PLINK2^27^ using weighted effects for each SNP. Before PRS/PGS calculation, SNPs were pruned using PLINK’s clump function with an r^2^ < 0.1 over 1000 kilo base pair (Kbp). The *APOE* gene is the most significant AD risk factor, with strong linkage disequilibrium (LD) levels in the locus region. Therefore, SNPs falling within the *APOE* gene region (chr19:44400000–46500000; GRCh37/hg19 assembly) were omitted from the dataset. The *APOE* ε2/ε3/ε4 status SNPs (rs7412 and rs429358) genotypes were then reintroduced into the dataset to ensure that the genetic risk of *APOE* was captured. Finally, the PRS / PGS was determined for each subject by summing up the adequate number of alleles (0, 1, 2) of the SNPs weighted by the natural logarithm of their respective ORs (Odds Ratio). The default formula for PRS calculation in PLINK is:

$$PRS_{j} = \frac{{\mathop \sum \nolimits_{i}^{N} S_{i} *G_{ij} }}{{P* M_{j} }}$$

where the effect size of SNP *i* is S_i_; the number of effect alleles observed in sample j is G_ij_; the ploidy of the sample is P (generally 2 for humans); the total number of SNPs included in the PRS is N, and the number of non-missing SNPs observed in sample j is M_j_. If the sample has a missing genotype for SNP i, then the population minor allele frequency multiplied by the ploidy (MAF_i_ ∗ P) is used instead of G_ij_^27^.

Publicly accessible summary statistics from reported GWAS studies (not overlapping with our dataset) of AD^12^, intelligence^13^ and educational attainment^14^ were used to define PRS for Alzheimer's, PGS for intelligence and educational attainment, respectively. We iterated over a variety of values (0.05 to 5 × 10^−8^) to evaluate the appropriate *p* value threshold *p* = 0.05 [PRS/PGS 1], *p* = 5 × 10^−3^ [PRS/PGS 2], *p* = 5 × 10^−4^ [PRS/PGS 3], *p* = 5 × 10^−5^ [PRS/PGS 4], *p* = 5 × 10^−6^ [PRS/PGS 5], *p* = 5 × 10^−7^ [PRS/PGS 6] and the GWAS-level significance thresholds of *p* = 5 × 10^−8^ [PRS/PGS 7] creating models named PRS/PGS 1–7.

### Aβ status

Cerebrospinal fluid (CSF) was analyzed for β-amyloid42 (Aβ42) and Aβ40 using Euroimmun (EI) immunoassay (EUROIMMUN AG, Lübeck, Germany). The Aβ status was defined as negative or positive using the CSF Aβ42/Aβ40 ratio, with Aβ+ defined as a ratio less than 0.09^28^.

### Bioinformatics analysis

We performed gene ontology (GO) enrichment analysis, protein–protein interaction (PPI) analysis and pathway analysis for the unique set of genes contributing to the PRS/PGS in each of the most significant PRS/PGS. We mapped each SNP to its nearest corresponding protein-coding gene (PCG) within one mega base pair (1 Mbp) of distance. If a SNP had no PCG within 1 Mbp, it was excluded from this analysis. We used Gonet^29^, STRING^30^ and Reactome database^31^ for the GO enrichment, PPI and pathway analysis, respectively.

### Identification of the Aβ independent PRS-Alz variants

To identify Aβ independent variants in the most significant PRS-Alz we applied a heuristic approach. First, we generated “n” different PRSs (where n = number of variants in the most significant PRS-Alz) using only “n − 1” variants of the most significant PRS-Alz and removing one specific variant in order from each of these “n” different PRSs. Next, for each of these reduced PRSs we tested if Aβ mediated the association between the reduced PRSs and rate of change in MMSE. Each of the “n” PRSs were ranked in ascending order of *p* value for the association between PRS and Aβ. Next, we again generated “n” different PRSs using this list of variants with each PRS consisting of ascending number of variants (e.g. the first PRS only has the first variant, the second PRS has the first top 2 variants, the third PRS has the first top 3 variants and so on) and again tested if Aβ mediated the association between the reduced PRSs and rate of change in MMSE. Finally, we ranked the variants by descending order of *p* value for the associations between PRS and Aβ, to identify Aβ dependent variants.

### Statistical analyses

Using the lmer function (lme4 package), mixed-effect models were fitted through maximum likelihood, using longitudinal MMSE data as the dependent variable. Random slopes and intercepts were extracted, rank-based inverse normal transformed (INT) and used as the dependent variable in linear regression models to test associations between PGS/PRS and cognition. The models were adjusted for age, gender, education, baseline MMSE (not for the intercept), *APOE* ε2 and ε4 count and the top 10 principal components (PC) from the principal component analysis (PCA) on the entire set of genotype data. Each set of association analyses was corrected family-wise error rate (FWER) using Bonferroni correction. All associations below Bonferroni corrected *p* value of 0.05 were considered significant. For mediation analysis, we tested the significance of the indirect effect using a bootstrap procedures (n = 1000 bootstrap samples were used to calculate the 95 percent confidence intervals of the effects). All the statistical analysis was conducted in R programming (version 4.0.2).

### Ethics approval

The Regional Ethics Committee in Lund, Sweden, approved the study. All subjects gave their written informed consent.

## Results

Table 1 shows the characteristics of the individuals and Figure S1 (supplementary file) shows subject-level data for longitudinal MMSE.

**Table 1:** Demographics and baseline characteristics.

	CU (Aβ −)	CU (Aβ +)	MCI (Aβ −)	MCI (Aβ +)	Total	*p* Value
N	260	121	50	127	558	
Female (%)	154 (59.2)	64 (52.9)	16 (32)	62 (48.8)	296 (57.1)	0.5
Age (years)	71.5 (5.3)	73.1 (4.7)	68.6 (5.4)	73 (5)	71.9 (5.2)	0.14
Education (years)	12.5 (3.5)	12.1 (3.9)	10.7 (3.2)	11.3 (3.3)	12 (3.6)	0.0003
*APOE* ε4 (0/1/2)	204/54/2	45/57/19	36/11/3	38/68/21	323/190/45	< 2e−16
*APOE* ε2 (0/1/2)	214/42/4	114/6/1	41/8/1	115/12/0	484/68/6	0.04
MMSE (IQR) (Median)	(30–29) (29)	(30–28) (29)	(29–26) (28)	(28–25) (27)	(30–27) (29)	< 2e−16

Age and education data are mean (standard deviation). *CU* cognitively unimpaired, *MCI* mild cognitive impaired. The group mean difference was calculated based on ANOVA.

### Alzheimer’s PRS and Intelligence PGS are associated with cognitive decline

**Figure 1:**
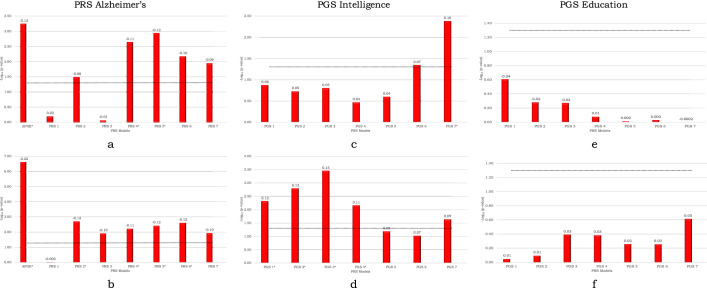
Associations of Polygenic Scores of AD, intelligence and education with cognition. (**a**) PRS-Alz association with the slope of longitudinal MMSE score. (**b**) PRS-Alz association with the intercept of MMSE score. (**c**) PGS-Int association with the slope of longitudinal MMSE score. (**d**) PGS-Int association with the intercept of MMSE score. (**e**) PGS-Edu association with the slope of longitudinal MMSE score. (**f**) PGS-Edu association with the intercept of MMSE score. The x-axis represents the 7 different PRS/PGS models at different *p* value thresholds based on the GWAS summary statistics (PRS1 ≤ 0.05, PRS2 ≤ 5e−3, PRS3 ≤ 5e−4, PRS4 ≤ 5e−5, PRS5 ≤ 5e−6, PRS6 ≤ 5e−7, PRS7 ≤ 5e−8). For PRS-Alz additional model was constructed with *APOE* alone (Represented on the x-axis as APOE). The models were adjusted for age, gender, education, baseline MMSE (not for the intercept), *APOE* ε2 and ε4 count and the top 10 principal components (PC) from the principal component analysis (PCA) on the entire set of genotype data. The y-axis shows the negative log of the *p* value for the significance of associations between PRS models with slope and intercept of longitudinal MMSE score. The values on the top of each bar show the effect size (beta-coefficient) of the association. The horizontal dotted line shows the *p* value threshold of 0.05. *These PRSs were significant after Bonferroni-correction at *p* value < 0.05.

We first tested effects of the three polygenic scores, i.e., PRS for AD (PRS-Alz), PGS for intelligence (PGS-Int) and educational attainment (PGS-Edu), on longitudinal cognitive decline. As explained above, we tested seven different levels of each score (e.g. PRS-Alz 1–7) with fewer SNPs for higher levels. PRS-Alz 4 and 5 (*p* value threshold 5 × 10^−5^ and 5 × 10^−6^ respectively) had significant associations with both lower intercept (Bonferroni corrected *p* values = 4.59e−02 and 2.9e−02 respectively) and steeper decline of slope (Bonferroni corrected *p* values = 1.8e−02 and 9.36e−03 respectively) for MMSE, meaning that the polygenic predictors were associated with lower MMSE score at baseline and accelerated cognitive decline. PRS-Alz 2 and 6 (*p* value threshold 5 × 10^−3^ and 5 × 10^−7^ respectively) were significantly associated with lower intercept (lower MMSE score at baseline, *p* = 1.56e−02 and *p* = 1.94e−02 respectively [Bonferroni corrected]) but not with the slope (*p* = 2.56e−01 and *p* = 5.38e−02 respectively [Bonferroni corrected]). PRS-Alz 1, 3 and 7 had no significant associations with intercept or slope of MMSE. This analysis was also tested using *APOE* alone as a predictor (without additional genetic data). We found a strong association of *APOE* with both lower intercept and slope of MMSE (*p* = 1.9e−06 and *p* = 4.53e−03, respectively [Bonferroni corrected]) (Fig. 1a,b, supplementary tables S1, S2).

For the PGSs of intelligence, PGS-Int 7 had a strong association with MMSE slope (*p* = 2.91e−02 [Bonferroni corrected]) but not with intercept (*p* = 1.59e−01 [Bonferroni corrected]). Multiple PGS-Int (PGS 1, PGS 2, PGS 3 and PGS 4) had significant associations with MMSE intercept (Fig. 1c,d, supplementary tables S3, S4). None of the PGS-Edu (Fig. 1e,f, supplementary tables S5a, S6a) was significantly associated with MMSE intercepts or slopes. We also repeated this analysis when excluding baseline education as a covariate, but there were still no significant associations between PGS-Edu and MMSE intercepts or slopes (supplementary tables S5b, S6b).

Further, we tested the association of the demographic covariates (age, gender, and education) with the slope and intercept of MMSE. We found steeper decline in cognition with higher age, less education, and male gender (supplementary table S7). We did not observe any significant association between baseline cognitive measure (intercept) and age or gender, but higher education was strongly associated with higher baseline cognitive measures (supplementary table S8).

As an exploratory analysis, we tested interactions between PRS-Alz 4, 5 and *APOE* and PGS-Int 7 (since these were significant in the analyses above) to predict MMSE slopes. None of the interactions was significant (*p* = 0.3, for PRS-Alz 4 × PGS-Int 7, *p* = 0.1, for PRS-Alz 5 × PGS-Int 7 and *p* = 0.9, for APOE × PGS-Int 7) (supplementary table S9) suggesting that APOE, and the polygenic contributions to AD and intelligence do not influence each other’s effect on cognitive decline.

### Significant PGS/PRS is associated with cognitive decline irrespective of Aβ-status

**Figure 2:**
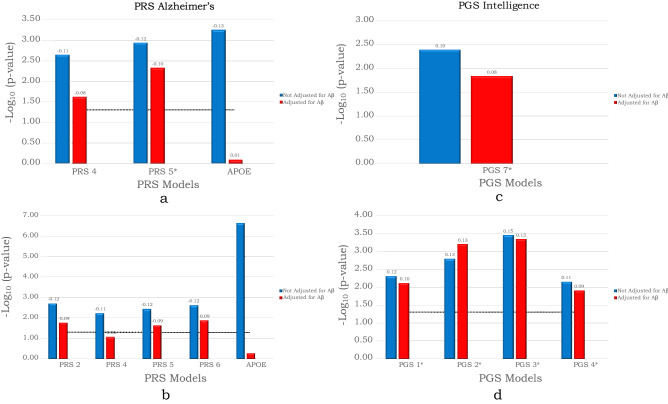
Associations of Polygenic Scores of AD and intelligence with cognitive decline after adjusting for Aβ-status. (**a**) PRS-Alz association with the slope of longitudinal MMSE score. (**b**) PRS-Alz association with the intercept of longitudinal MMSE score. (**c**) PGS-Int association with the slope of longitudinal MMSE score. (**d**) PGS-Int association with the intercept of longitudinal MMSE score. PRS/PGS models that showed significant associations with MMSE in base models (when not adjusted for Aβ-status) are included and displayed on the x-axis. The y-axis shows the negative log of the *p* value for the significance of associations between PRS models with a random slope and random intercept of longitudinal MMSE score. The values on the top of each bar show the effect size (beta-coefficient) of the association. The horizontal dotted line shows the *p* value threshold of 0.05. *These PRSs were significant after Bonferroni-correction at *p* value < 0.05.

The models described above were not adjusted for Aβ-status. To test if the effects of the identified PGS/PRSs on cognition depended on Aβ-positivity (indicating underlying AD pathology), we tested the significant PRG/PRSs with the additional adjustment for Aβ status. This attenuated the associations, but PRS-Alz 5 still showed a significant association with the slope of longitudinal MMSE after Bonferroni correction (*p* = 1.42e−02). The association between the model with APOE alone and the slope of MMSE was non-significant when adjusting for Aβ status (*p* = 0.8) and there was a considerable attenuation in the effect size (Fig. 2a, supplementary table S10). None of the PRS-Alz or APOE models showed any significant association with the random intercept after Bonferroni correction (Fig. 2b, supplementary table S11). Taken together, these results suggests that the effect of *APOE* on cognitive decline largely depends on Aβ status (while the polygenic risk for AD has some effects on cognition that are independent of Aβ pathology).

After adjusting for Aβ status, PGS-Int 7 remained significantly associated with the slope of MMSE (*p* = 1.49e−02) (Fig. 2c, supplementary table S12) and PGS-Int 1, 2, 3 and 4 with the intercept (*p* = 3.06e−02, 2.55e−02, 1.86e−02 and 4.96e−02 respectively [Bonferroni corrected]) (Fig. 2d, supplementary table S13). This suggests that polygenic contributors to intelligence contribute to cognition largely independent of Aβ pathology.

### No interaction between PRS/PGS association and Aβ-status for cognitive decline

To further test if the effects were specific to an Aβ subgroup (positive or negative), we next included an interaction term for Aβ status and PRS/PGS in the model. The interaction term was non-significant for all PRS/PGS models. For the APOE model, the interaction term was significant for the intercept (uncorrected *p* value = 0.04) but not for the slope of MMSE (supplementary tables S14–S19).

### Mediation analyses

**Figure 3:**
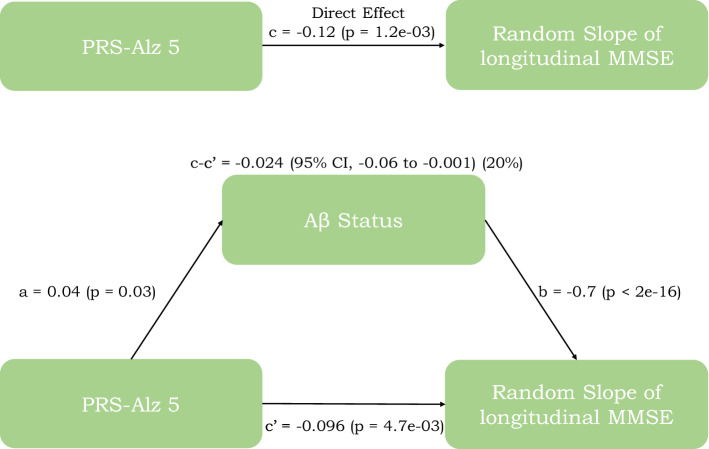
Mediation analysis between PRS, Aβ status and cognition. Mediation analysis with PRS-Alz 5 (predictor), mediated by Aβ status predicting the cognitive decline (slope of longitudinal MMSE). The figure includes the following standardized regression coefficients: a, the effects of PRS/PGS on Aβ; b, the effects of Aβ on the random slope of longitudinal MMSE score; c, the direct association between PRS and slope of longitudinal MMSE score; c’, the association between PRS and slope of longitudinal MMSE score when adjusting for Aβ; and c–c’, the mediated effect on the slope of longitudinal MMSE (with % mediation).

As described above, we observed a significant effect of PRS/PGS on cognitive decline when not adjusting for Aβ-status, and attenuated effects when adjusting for Aβ-status. We next tested associations between the significant PRS/PGS and Aβ-status. PRS-Alz 5 showed a significant association with Aβ-status (*p* = 0.03), but this was not seen for PGS-Int 7 and PGS-Int 3 (*p* = 0.08 and *p* = 0.3 respectively). Given these findings, we performed a mediation analysis for PRS-Alz 5 to quantify the extent to which Aβ mediated the effects of the PRS on cognitive decline. Figure 3 illustrates that the rate of change of MMSE that is significantly predicted by PRS-Alz 5 is partly but significantly mediated by Aβ pathology (20% mediation effect) (supplementary table S20).

### PRS-variants independent of Aβ

The results above suggested that the effect of PRS-Alz 5 on longitudinal cognition was significantly but only partly mediated by Aβ pathology. We hypothesized that this PRS might be heterogeneous and contain some genetic components that had an effect on cognition through accumulation of Aβ pathology, and others that were truly independent of Aβ. Using a heuristic approach (method section for details), we set out to disentangle these components, by testing different subsets of PRS-Alz 5. We identified 16 variants that might be independent of Aβ, as the *p* value of association between the pruned PRS and Aβ was reduced (indicating a more significant association that in the original PRS-Alz 5) when these variants were removed from the PRS. We also found 17 variants whose removal resulted in an increase in the *p* value of association of PRS with Aβ relative to PRS-Alz 5 (indicating a less significant association), suggesting that these variants may be Aβ-dependent (supplementary table S21). Among the different PRSs, we could identify an Aβ-independent PRS model (hereafter called PRS-Alz 5-I, where I stand for independent), which consisted of the top 23 variants to be the best model independent of Aβ status. For PRS-Alz 5-I, there was no mediation effect shown by Aβ, but there was still a significant effect on cognitive decline, both when not adjusting the model for Aβ (beta =  − 0.07, *p* = 4.9 e−02) and when adjusting for Aβ (beta =  − 0.08, *p* = 1.5e−02) (more extensive PRSs did not meet these conditions). Taken together, these findings support that a PRS consisting of these 23 variants is Aβ-independent (supplementary table S22). To test for the Aβ dependent set of variants, we analyzed these variants by ranking them in the reverse order. The PRS model (hereafter called as PRS-Alz 5-D, where D stand for dependent) with the top 10 variants (reverse order ranking) was the best Aβ-dependent model, as the association between the PRS model and slope of MMSE was lost when adjusting for Aβ (beta =  − 0.06, *p* = 8.5e−02) compared to when not adjusting for Aβ (beta =  − 0.11, *p* = 2e−03) (supplementary table S23) (this condition was not met for more extensive PRSs). Among these ten variants, three variants (rs3851179, rs11257240 and rs9649710) made the Aβ adjusted effect significant when added in the models. We tested the models by removing these SNPs (one at a time) and found that even after removal of rs11257240 and rs9649710 the model not adjusted for Aβ remained significant (beta =  − 0.09, *p* = 1.5e−02) and the model adjusted for Aβ was non-significant (beta =  − 0.04, *p* = 0.2). These findings suggested that eight of the ten variants are dependent on Aβ whereas reaming two variants may be partially dependent on Aβ. To further confirm the variants that were dependent on Aβ we tested the individual associations between each SNP and Aβ. We found two SNPs (rs3851179 and rs6733839) to be significantly associated with Aβ (uncorrected *p* value = 2.6e−02 and 2.4e−02 respectively). The remaining eight SNPs from the Aβ dependent variant set had strong effect sizes (beta > 0.15) when predicting Aβ, but were non-significant (possibly due to insufficient power) (supplementary table S24).

### Bioinformatics analysis of the most significant PRS/PGS

**Figure 4:**
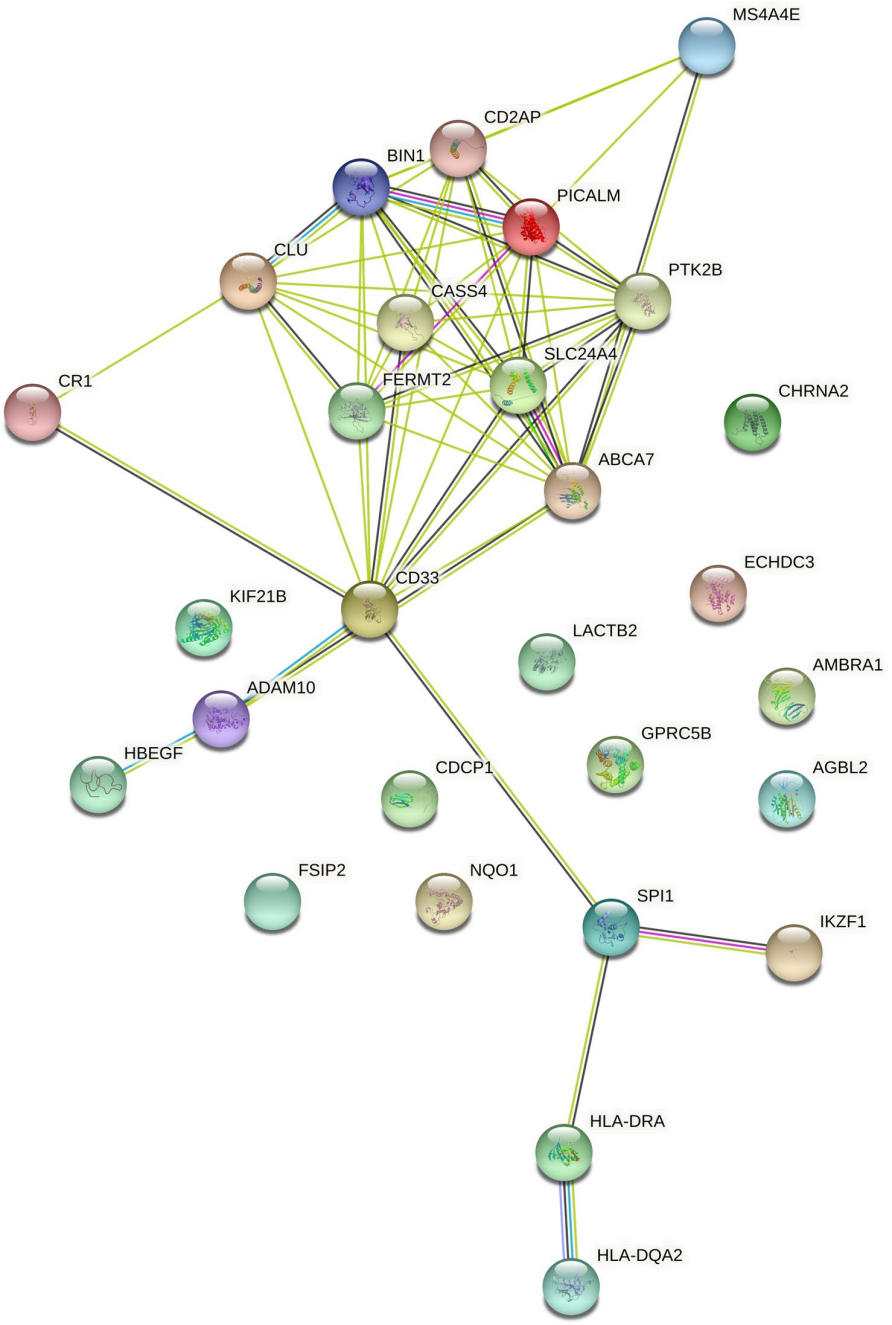
Bioinformatics analysis of Alzheimer’s disease PRS-Alz 5: Protein–Protein Interaction network for the protein-coding genes of the SNPs of PRS-Alz 5 (PPI enrichment *p* value: < 1.0e−16). Nodes represent the proteins and edges indicate both functional and physical protein association. The edge line thickness shows the strength of data support at an interaction score ≥ 0.4.

**Figure 5:**
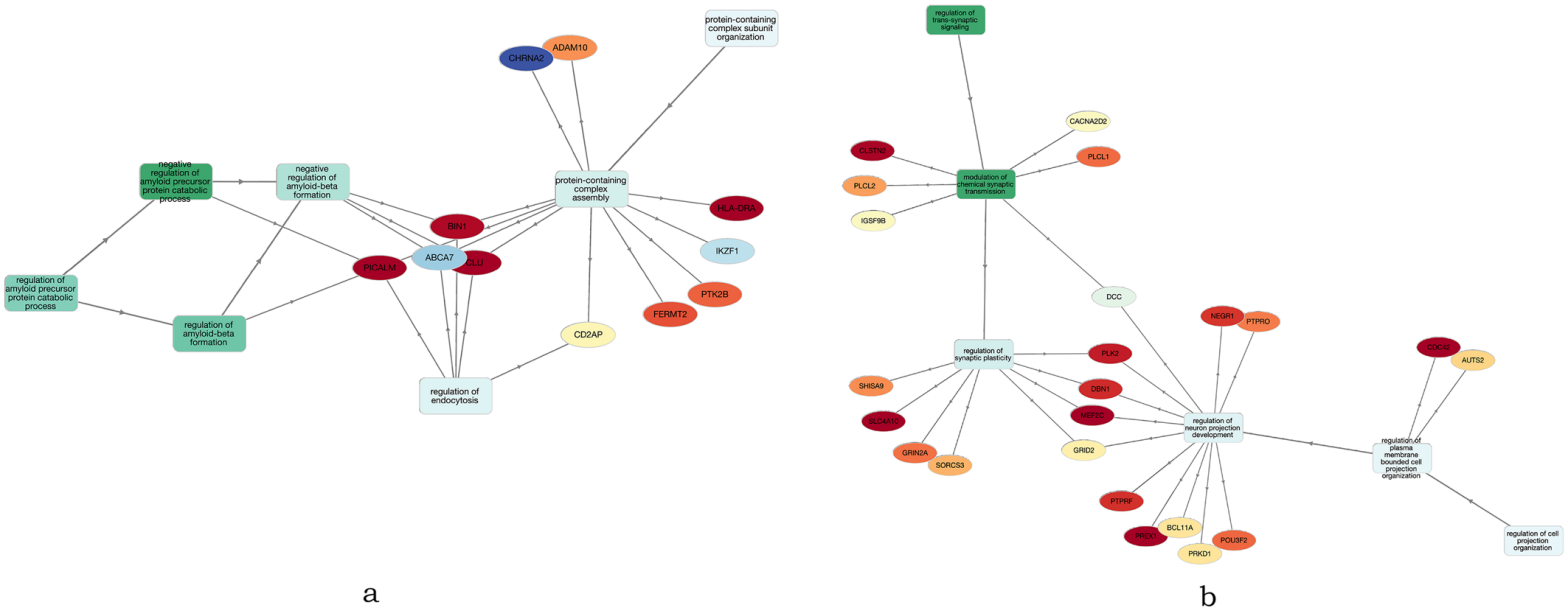
Gene ontology term enrichment of genes of the variants involved in (**a**) PRS-Alz 5 and (**b**) PGS-Int 7 for biological process category. Oval shaped boxes represent the protein-coding genes and the rectangular boxes define the GO term for the biological category. The oval boxes are coloured based on protein expression in the cerebral cortex as per the Human protein Atlas dataset.

To elucidate the biological pathways that contributed to altered risk for cognitive decline, we performed bioinformatics analyses of the key PRS (PRS-Alz 5) and PGS (PGS-Int 7) results that were associated with cognitive decline. Thirty-three SNPs of “PRS-Alz 5” mapped to 28 unique PCG (supplementary table S25). Out of these 28, ten PCGs did not show any interaction with other PCGs at an interaction score of 0.4 (medium confidence)^23^ (figure S2). Figure 4 shows the PPI network of the 18 PCGs enriched at *p* value < 1e−16. The presence of such enrichment implies that the proteins are at least partially biologically connected as a group. The GO enrichment analysis identified four genes (*ABCA7*, *BIN1*, *CLU* and *PICALM*) which are involved in the regulation of Aβ formation as the most top GO terms in the Biological Process category (*p* value ≤ 1.98e−03 [FDR corrected]) (Fig. 5a, supplementary table S26). In the pathway analysis, we found no significant pathway enrichment for the PCGs after the FDR correction a *p* < 0.05. The top pathway enrichment term was “clathrin-mediated endocytosis pathway”, which involved *BIN1*, *PTK2B*, *PICALM* and *HBEGF* (9.85e−02 [FDR corrected]) (supplementary table S27).

For the intelligence PGS, we found that 176 SNPs of “PGS-Int 7” were mapped to 170 unique PCGs (supplementary table S28). Making a PPI network with such a large number of the gene set was beyond the scope of this study. For “PGS-Int 7” GO enrichment analysis returned 6 GO terms enriched for the 170 PCGs at FDR corrected *p* value < 0.05. “modulation of chemical synaptic transmission” was the top term in this category (*p* = 6.26e−04 [FDR corrected]) followed by three other terms related to “neuronal activity” (“regulation of trans-synaptic signalling”, “regulation of synaptic plasticity” and “regulation of neuron projection development”) for the biological process at *p* ≤ 4.26e−02 (FDR corrected) (Fig. 5b, supplementary table S29). The pathway enrichment analysis identified only one term, “MECP2 regulates transcription factors”, as the top category with FDR corrected *p* value = 6.94e−03 (supplementary table S30).

### PRS/PGS association analysis with other cognitive measures as an outcome

We used MMSE as our main outcome since it is both widely used and well standardized. To further strengthen our findings, we performed additional analysis by using four other cognitive measures: ADAS-Cog delayed recall test, AQT, TMT-B and PACC. PRS-Alz 2, 3, 4 and 5 showed a strong association with the slope of ADAS-Cog delayed recall test (Bonferroni corrected *p* values = 5.5e−03, 1.04e−02, 2.5e−02 and 2.9e−02 respectively). PRS-Alz 6 showed a significant association with the slope of AQT (supplementary table S31 and supplementary figure S3a). We also found PRS-Alz 4, 5 and 6 showing significant associations with the intercept of ADAS-Cog delayed recall test (Bonferroni corrected *p* values = 9.6e−03, 1.9e−02 and 4.5e−02 respectively) and with the intercept of PACC (Bonferroni corrected *p* values = 6.7e−03, 5.4e−03 and 5.4e−03 respectively). PRS-Alz 7 and PRS-Alz 4 showed significant associations with the intercept of PACC (Bonferroni corrected *p* value = 4.8e−03) and TMT (Bonferroni corrected *p* value = 3.3e−02) respectively (supplementary table S32 and supplementary figure S3b).

For PGS-Int, we found PGS 1 showing a significant association with TMT slope (Bonferroni corrected *p* value = 4.9e−02) (supplementary table S33 and supplementary figure S4a). The intercept of ADAS-cog delayed recal and AQT showed a significant association with PGS 2 (Bonferroni corrected *p* value = 1.7e−02) and PGS 1 (Bonferroni corrected *p* value = 2.3e−03) respectively (supplementary table S34 and supplementary figure S4b). However, no significant association was found between the PGS-Edu and slope or intercept of the four cognitive measures (supplementary tables S35, S36 and supplementary figures S5a and b).

## Discussion

In this study of genetic contributions to cognitive decline in the earliest stages of AD, we investigated whether a priori defined PRS/PGS for AD, intelligence and educational attainment were associated with cognitive decline in CU and MCI individuals depending upon their Aβ status. Our main findings were that the PRS for AD was related to the rate of cognitive decline and was partly mediated through Aβ-pathology, while an intelligence PGS was protective in CU and MCI participants irrespective of Aβ status. These findings suggest that a priori defined genetic risk factors for AD may influence the disease by affecting the rate of cognitive decline for people with early AD. In contrast, genetic factors linked to intelligence may affect the overall rate of decline in cognition. In addition, it is possible that treatments that mimic or interfere with specific biological pathways identified through these genetic analyses may have the potential to affect the rate of disease progression in early-stage AD.

We also tested a PRS model with *APOE* alone as a predictor, which had substantial effects on cognition. However, the strong effect of *APOE* was lost entirely when the analysis was adjusted for Aβ status, indicating that the cognitive decline was associated more closely via Aβ pathology rather than *APOE*. The significant interaction effect between APOE and Aβ with the intercept of MMSE suggested that the Aβ positive individuals with APOE had the most marked cognitive loss at the start of the study, although the non-significant interaction for the slope of MMSE indicated that APOE did not affect rate of progression once accounting for Aβ status.

Notably, in our exploratory analysis of individual genetic variants and rate of cognitive decline adjusting with and without *APOE* ε4 burdens resulted in the same variant rs10492328 as a top hit with similar p-value threshold (*p* = 4.4e−07) (supplementary results, supplementary tables S37, S38). This finding suggests that in the early stages of AD, the genetic control of the rate of cognitive decline is mainly unrelated to *APOE,* once taking Aβ burden into account (although the age of onset of AD is clearly affected by APOE genotype^32^). This agrees with our recent finding that effects of Aβ-burden on cognition does not vary by *APOE* genotype in very early stages of AD^33^

Previous studies of genetic risk factors for cognitive decline in early stages of AD using the Alzheimer’s Disease Neuroimaging Initiative (ADNI) subjects^34^ and Australian Imaging, Biomarkers and Lifestyle (AIBL)^35^ found significant effects of *APOE* ɛ4 and PGSs associated with AD^34,35^. A study focused on preclinical AD subjects from the AIBL study used episodic memory PRS and predicted rates of cognitive decline in domains typically affected in the preclinical stages of AD^36^. Unlike those studies^34–36^, our findings suggest that a well-defined AD PRS can predict cognitive decline over and above *APOE* ɛ4 at an early stage of AD. This is consistent with one study that used polygenic hazard score (PHS) over and above *APOE* ɛ4 to predict the longitudinal clinical decline in older individuals with moderate to high amyloid load^37^. Thus, such PRSs may potentially be used (together with other modalities) to improve the early prognostics of risk individuals. Looking for the PRS effect on the early disease process, another recent study showed the association of AD PRS with an increased probability of MCI compared to normal individuals at 50 years of age^38^. Perhaps more importantly, these genetic results may help to understand the biological processes of AD in early stages, as discussed further below.

The intelligence PGS-Int 7 was protective in both early-stage AD and in CU and MCI participants independent of Aβ status. This suggests that a high cognitive reserve might be associated with lower dementia risk. One possible explanation for this could be that individuals with higher ‘intelligence’ have a high level of cognitive ability, which may make them reach dementia diagnosis thresholds later than those with lower ‘intelligence’. It suggests that the genetic background that contributes to intelligence does not guard specifically against the development of AD, but rather delays the onset of symptoms from neurodegenerative diseases. Four intelligence PGS (PGS 1, PGS 2, PGS 3 and PGS 4) were only significantly associated with baseline cognition. The involvement of these PGSs in cognitive decline is uncertain. Previous studies have reported different results for associations between intelligence and AD. For example, one study found that intellectual enrichment was not a significant predictor of Aβ or AD-pattern neurodegeneration^39^. Two other studies found that intellectual enrichment may have marginal effects on AD biomarkers but a more substantial impact on delaying the onset of cognitive impairment^40,41^. Higher intelligence (and perhaps relevant genetic variants) was also associated with a lower risk of AD in a twin study^42^. Taken together, these and our findings support a model where genetic factors that contribute to higher intelligence are protective against general cognitive decline and may delay the onset of symptoms of AD (despite not affecting the underlying biological processes of the disease). However, further research is needed to prove this hypothesis.

Interestingly the PGS-Int 7 consist of all the SNPs that were significant at a genome-wide significant threshold of *p* < 5e−08. Similarly, for PRS-Alz 5, mapping the variants to unique PCG resulted in all known genome-wide significant variants reported in previous work^12^. Hence, it suggests that the significant effect in the PRS for AD and PGS for intelligence is reached when the PRS/PGS contains genome-wide significant variants.

There was no effect of education PGS, arguing against associations between educational attainment and rate of cognitive decline. This is in line with another recent study using educational attainment PGS to predict the rate of cognitive decline in non-demented individuals^43^. In addition, another recent research agreeing with our findings showed that education and cognitive function in midlife did not affect long-term brain Aβ accumulation^44^. Though we did not find any association between PGS-Edu and any of the cognitive scores, we did find the expected association between education in itself and cognitive decline (supplementary tables S7 and S8). Furthermore, in a supplemental analysis, we found PGS-Edu to be very strongly associated with baseline education (as expected). Taken together, our results indicate that education can still be a proxy for cognitive reserve (since we found that education as a covariate was associated with cognition in general in our models). Still, the genetic background that affects education is not sufficiently strongly associated with cognition to be used as a proxy for cognitive reserve.

The effect of the PRS-Alz 5 on cognitive decline was partly mediated by Aβ pathology, indicating a symbiotic effect between Aβ pathology and genetic factors related to AD. Mediation analysis shows that around 20% effect of PRS-Alz on cognitive decline was mediated by Aβ pathology (both at baseline and progression). This finding is consistent with the AD pathological cascade, suggesting that Aβ biomarkers become abnormal long before cognitive decline during the development of AD^45^. While Aβ levels become pathological long before extreme cognitive impairments manifest, new research^46,47^ indicates that subtle cognitive changes can begin early, perhaps before Aβ reaches the abnormality threshold. Early intervention could be significantly aided by identifying at-risk patients before Aβ hits pathological thresholds.

Dividing PRS-Alz 5 into Aβ dependent (PRS-Alz-D) and independent component (PRS-Alz-I) identified eight variants to be Aβ dependent, two variants partially dependent of Aβ and 23 variants independent of Aβ. Mapping these variants to genes resulted in 21 unique genes (CD33, ABCA7, ADAM 10 to name few, refer supplementary table S22 for full list) for the PRS-Alz-I variants and eight for PRS-Alz-D (PICALM, CR1 among the top genes, refer supplementary table S23). Among these except for *NIFKP9,* all other genes were specific to either Aβ independent or dependent groups. *NIFKP9* is a pseudogene around 80 Mbp away from *BIN1* and is falling in both Aβ independent (rs6710467) dependent (rs6733839) category. The two variants possibly work opposite to each other, as evident from our association analysis of individual variants with Aβ (supplementary table S24). rs6733839 (beta = 0.23, *p* = 2.4e−02) has a significantly stronger association with Aβ compared to rs6710467 (beta =  − 0.02, *p* = 8.7e−01). Pseudogenes modulate the expression of the functional genes^48^, and studies have reported that any sequence variation in the pseudogenes will have serious implications on the expression of the functional gene^49^. Taken together with these findings, it is possible that different variants within *NIFKP9* may regulate Aβ both positively and negatively.

Bioinformatics analysis based on the GO enrichment analysis showed that most of the genes of PRS-Alz 5 were involved with the regulation of Aβ in the brain. The lower enrichment p-value indicates that the proteins are at least partially biologically connected and associated with the rate of cognitive decline in harmony. The pathway analysis showed “Clathrin-mediated endocytosis” as the top pathway hit (non-significant after FDR correction) in which the significant genes (*BIN1, PTK2B, PICALM, HBEGF*) of the AD PRS are involved. Previous studies have shown that this pathway plays a central role in the production of Aβ in neurons^50,51^. Our pathway analysis also showed Trges pathway as a second top hit. Dysfunction of Tregs has been associated with neurodegenerative diseases, such as AD^52^.

For the Intelligence PGS-Int 7, GO enrichment terms related to synaptic signaling were the top hits. Previous research has identified that the genes involved in such synaptic signaling pathways play an essential role in cognitive ability^53^ and disruption of such pathways may result in cognitive deficit^54^. The pathway analysis the “MECP2 regulates transcription factors” pathways to be top hit, where *MECP2* is involved in the regulation of gene activity. MeCP2 protein is vital for the function of several types of cells, including nerve cells. The genetic loss of *MECP2* has been identified as changing the properties of cells in the locus coeruleus (the exclusive source of noradrenergic innervation to the cerebral cortex and hippocampus)^55^.

## Limitations

Although the BioFINDER cohort has extensive phenotyping for cognition and Aβ that is necessary for a study of early-stage AD, the sample size was relatively small. We, therefore, restricted the analysis to genes with MAF ≥ 0.05. More extensive studies would be necessary to consider the rarer genetic variants, which could also be essential for cognitive decline. A higher frequency of testing and longer follow up data must be collected to validate further and optimize these genetic risk predictors. Another limitation is that several brain changes may contribute to cognitive decline in the elderly. Our study was only adjusted for Aβ. Follow-up studies in larger samples adjusting for both Aβ and tau may be done to better clarify the genetic influence on cognition (since the combination of tau and Aβ pathology may be crucial for accelerated cognitive decline). Future studies may also adjust for other brain changes, e.g. vascular pathology and synucleinopathy which may also contribute to cognitive decline. Such more comprehensive adjustments may sharpen the association between cognition and PRSs.

## Conclusions

The study evaluated the rate of cognitive decline based on PRS of AD and PGS of intelligence and education. PRS-Alz was associated with rate of cognitive decline and was partly mediated by Aβ, while PGS-Int was protective irrespective of Aβ status. There was no effect of PGS-Edu in any of the populations. Novel genetic associations with the rate of cognitive decline in AD may provide new insights into the pathophysiology of AD and suggest new therapeutic development targets.

## Supplementary Information

Supplementary Information 1.

Supplementary Information 2.

## Data Availability

The summary statistics of the BIOFINDER GWAS is available from the corresponding author on reasonable request. Genome-wide summary statistics used to generate Alzheimer’s PRS can be downloaded from the National Institute on Aging Genetics of Alzheimer’s Disease Data Storage Site (NIAGADS)—an NIA/NIH-sanctioned qualified-access data repository, under accession NG00075. Summary statistics used for the generation of Intelligence PRS are available for download from https://ctg.cncr.nl/. Summary statistics used for the generation of educational attainment PRS can be downloaded from http://www.thessgac.org/data.

## Abbreviations

AD: Alzheimer’s disease
PGS: Polygenic scores
GWAS: Genome-wide association study
MMSE: Mini-mental state examination
Aβ: β-Amyloid
CU: Cognitively unimpaired
MCI: Mild cognitive impairment
PRS: Polygenic risk score
SCD: Subjective cognitive decline
QC: Quality control
HWE: Hardy–Weinberg equilibrium
MAF: Minor allele frequency
LD: Linkage disequilibrium
CSF: Cerebrospinal fluid
GO: Gene ontology
PPI: Protein–Protein interaction

## References

[1] Perrault A, Wolfson C, Egan M, Rockwood K, Hogan DB (2002). Prognostic factors for functional independence in older adults with mild dementia: Results from the Canadian study of health and aging. Alzheimer Dis. Assoc. Disord..

[2] Holmes C, Lovestone S (2003). Long-term cognitive and functional decline in late onset Alzheimer’s disease: therapeutic implications. Age Ageing.

[3] Corey-Bloom J, Fleisher AS (2000). The natural history of Alzheimer’s disease. Dementia.

[4] Holmes C (2002). Genotype and phenotype in Alzheimer’s disease. Br. J. Psychiatry.

[5] Lo MT, Kauppi K, Fan CC, Sanyal N, Reas ET, Sundar VS, Lee WC, Desikan RS, McEvoy LK, Chen CH (2019). Alzheimer’s disease genetics consortium. Identification of genetic heterogeneity of Alzheimer’s disease across age. Neurobiol. Aging.

[6] Holmes C, Ballard C, Lehmann D, Smith AD, Beaumont H, Day IN, Khan MN, Lovestone S, McCulley M, Morris CM, Munoz DG (2005). Rate of progression of cognitive decline in Alzheimer’s disease: Effect of butyrylcholinesterase K gene variation. J. Neurol. Neurosurg. Psychiatry.

[7] Fan J, Tao W, Li X, Li H, Zhang J, Wei D, Chen Y, Zhang Z (2019). The contribution of genetic factors to cognitive impairment and dementia: Apolipoprotein E gene, gene interactions, and polygenic risk. Int. J. Mol. Sci..

[8] Euesden J, Gowrisankar S, Qu AX, Jean PS, Hughes AR, Pulford DJ (2020). Cognitive decline in Alzheimer’s disease: Limited clinical utility for GWAS or polygenic risk scores in a clinical trial setting. Genes.

[9] Reitz C, Mayeux R (2010). Use of genetic variation as biomarkers for mild cognitive impairment and progression of mild cognitive impairment to dementia. J. Alzheimer’s Dis..

[10] Bäckman L, Jones S, Small BJ, Agüero-Torres H, Fratiglioni L (2003). Rate of cognitive decline in preclinical Alzheimer’s disease: The role of comorbidity. J. Gerontol. B Psychol. Sci. Soc. Sci..

[11] Lee E, Giovanello KS, Saykin AJ, Xie F, Kong D, Wang Y, Yang L, Ibrahim JG, Doraiswamy PM, Zhu H (2017). Alzheimer’s disease neuroimaging initiative. Single-nucleotide polymorphisms are associated with cognitive decline at Alzheimer’s disease conversion within mild cognitive impairment patients. Alzheimer’s Dementia Diagn. Assess. Dis. Monit..

[12] Kunkle BW, Grenier-Boley B, Sims R, Bis JC, Damotte V, Naj AC, Boland A, Vronskaya M, Van Der Lee SJ, Amlie-Wolf A, Bellenguez C (2019). Genetic meta-analysis of diagnosed Alzheimer’s disease identifies new risk loci and implicates Aβ, tau, immunity and lipid processing. Nat. Genet..

[13] Savage JE, Jansen PR, Stringer S, Watanabe K, Bryois J, De Leeuw CA, Nagel M, Awasthi S, Barr PB, Coleman JR, Grasby KL (2018). Genome-wide association meta-analysis in 269,867 individuals identifies new genetic and functional links to intelligence. Nat. Genet..

[14] Lee JJ, Wedow R, Okbay A, Kong E, Maghzian O, Zacher M, Nguyen-Viet TA, Bowers P, Sidorenko J, Linnér RK, Fontana MA (2018). Gene discovery and polygenic prediction from a 1.1-million-person GWAS of educational attainment. Nature Genet..

[15] Torkamani A, Wineinger NE, Topol EJ (2018). The personal and clinical utility of polygenic risk scores. Nat. Rev. Genet..

[16] Chasioti D, Yan J, Nho K, Saykin AJ (2019). Progress in polygenic composite scores in Alzheimer’s and other complex diseases. Trends Genet..

[17] Martin AR, Daly MJ, Robinson EB, Hyman SE, Neale BM (2019). Predicting polygenic risk of psychiatric disorders. Biol. Psychiat..

[18] Mattsson N, Insel PS, Palmqvist S, Stomrud E, Van Westen D, Minthon L, Zetterberg H, Blennow K, Hansson O (2016). Increased amyloidogenic APP processing in APOE ɛ4-negative individuals with cerebral β-amyloidosis. Nat. Commun..

[19] Ossenkoppele R, Rabinovici GD, Smith R, Cho H, Schöll M, Strandberg O, Palmqvist S, Mattsson N, Janelidze S, Santillo A, Ohlsson T (2018). Discriminative accuracy of [18F] flortaucipir positron emission tomography for Alzheimer disease vs other neurodegenerative disorders. JAMA.

[20] Jack CR, Bennett DA, Blennow K, Carrillo MC, Dunn B, Haeberlein SB, Holtzman DM, Jagust W, Jessen F, Karlawish J, Liu E (2018). NIA-AA research framework: toward a biological definition of Alzheimer’s disease. Alzheimer’s Dement..

[21] Folstein MF, Folstein SE, McHugh PR (1975). “Mini-mental state”: a practical method for grading the cognitive state of patients for the clinician. J. Psychiatr. Res..

[22] Rosen WG, Mohs RC, Davis KL (1984). A new rating scale for Alzheimer's disease. Am. J. Psychiatry..

[23] Kvitting AS, Wimo A, Johansson MM, Marcusson J (2013). A Quick test of cognitive speed (AQT): Usefulness in dementia evaluations in primary care. Scand. J. Prim. Health Care.

[24] Insel PS, Weiner M, Mackin RS, Mormino E, Lim YY, Stomrud E, Palmqvist S, Masters CL, Maruff PT, Hansson O, Mattsson N (2019). Determining clinically meaningful decline in preclinical Alzheimer disease. Neurology.

[25] Donohue MC, Sperling RA, Petersen R, Sun CK, Weiner MW, Aisen PS (2017). Alzheimer’s Disease Neuroimaging Initiative. Association between elevated brain amyloid and subsequent cognitive decline among cognitively normal persons. JAMA.

[26] Anderson CA, Pettersson FH, Clarke GM, Cardon LR, Morris AP, Zondervan KT (2010). Data quality control in genetic case-control association studies. Nat. Protoc..

[27] Chang CC, Chow CC, Tellier LC, Vattikuti S, Purcell SM, Lee JJ (2015). Second-generation PLINK: Rising to the challenge of larger and richer datasets. Gigascience.

[28] Janelidze S, Zetterberg H, Mattsson N, Palmqvist S, Vanderstichele H, Lindberg O, van Westen D, Stomrud E, Minthon L, Blennow K (2016). Swedish BioFinder Study Group. CSF Aβ42/Aβ40 and Aβ42/Aβ38 ratios: Better diagnostic markers of Alzheimer disease. Ann. Clin. Transla. Neurol..

[29] Pomaznoy M, Ha B, Peters B (2018). GOnet: A tool for interactive Gene Ontology analysis. BMC Bioinform..

[30] Szklarczyk D, Gable AL, Lyon D, Junge A, Wyder S, Huerta-Cepas J, Simonovic M, Doncheva NT, Morris JH, Bork P, Jensen LJ (2019). STRING v11: Protein–protein association networks with increased coverage, supporting functional discovery in genome-wide experimental datasets. Nucleic Acids Res..

[31] Jassal B, Matthews L, Viteri G, Gong C, Lorente P, Fabregat A, Sidiropoulos K, Cook J, Gillespie M, Haw R, Loney F (2020). The reactome pathway knowledgebase. Nucleic Acids Res..

[32] Liu CC, Kanekiyo T, Xu H, Bu G (2013). Apolipoprotein E and Alzheimer disease: Risk, mechanisms and therapy. Nat. Rev. Neurol..

[33] Insel PS, Hansson O, Mattsson-Carlgren N (2021). Association between apolipoprotein E ε2 vs ε4, age, and β-amyloid in adults without cognitive impairment. JAMA Neurol..

[34] Ge T, Sabuncu MR, Smoller JW, Sperling RA, Mormino EC (2018). Alzheimer’s disease neuroimaging initiative dissociable influences of APOE ε4 and polygenic risk of AD dementia on amyloid and cognition. Neurology.

[35] Porter T, Burnham SC, Milicic L, Savage G, Maruff P, Lim YY, Li QX, Ames D, Masters CL, Rainey-Smith S, Rowe CC (2018). Utility of an Alzheimer’s Disease risk-weighted polygenic risk score for predicting rates of cognitive decline in preclinical alzheimer’s disease: A prospective longitudinal study. J. Alzheimer’s Dis..

[36] Porter T, Burnham SC, Savage G, Lim YY, Maruff P, Milicic L, Peretti M, Ames D, Masters CL, Martins RN, Rainey-Smith S (2018). A polygenic risk score derived from episodic memory weighted genetic variants is associated with cognitive decline in preclinical Alzheimer’s disease. Front. Aging Neurosci..

[37] Tan CH, Hyman BT, Tan JJ, Hess CP, Dillon WP, Schellenberg GD, Besser LM, Kukull WA, Kauppi K, McEvoy LK, Andreassen OA (2017). Polygenic hazard scores in preclinical Alzheimer disease. Ann. Neurol..

[38] Logue MW, Panizzon MS, Elman JA, Gillespie NA, Hatton SN, Gustavson DE, Andreassen OA, Dale AM, Franz CE, Lyons MJ, Neale MC (2019). Use of an Alzheimer’s disease polygenic risk score to identify mild cognitive impairment in adults in their 50s. Mol. Psychiatry.

[39] Vemuri P, Knopman DS, Lesnick TG, Przybelski SA, Mielke MM, Graff-Radford J, Murray ME, Roberts RO, Vassilaki M, Lowe VJ, Machulda MM (2017). Evaluation of amyloid protective factors and Alzheimer disease neurodegeneration protective factors in elderly individuals. JAMA Neurol..

[40] Vemuri P, Lesnick TG, Przybelski SA, Machulda M, Knopman DS, Mielke MM, Roberts RO, Geda YE, Rocca WA, Petersen RC, Jack CR (2014). Association of lifetime intellectual enrichment with cognitive decline in the older population. JAMA Neurol..

[41] Vemuri P, Lesnick TG, Przybelski SA, Knopman DS, Machulda M, Lowe VJ, Mielke MM, Roberts RO, Gunter JL, Senjem ML, Geda YE (2016). Effect of intellectual enrichment on AD biomarker trajectories: Longitudinal imaging study. Neurology.

[42] Osler M, Christensen GT, Garde E, Mortensen EL, Christensen K (2017). Cognitive ability in young adulthood and risk of dementia in a cohort of Danish men, brothers, and twins. Alzheimer’s Dement.

[43] Kauppi K, Rönnlund M, Adolfsson AN, Pudas S, Adolfsson R (2020). Effects of polygenic risk for Alzheimer’s disease on rate of cognitive decline in normal aging. Transl. Psychiatry.

[44] Rawlings AM, Sharrett AR, Mosley TH, Wong DF, Knopman DS, Gottesman RF (2019). Cognitive reserve in midlife is not associated with amyloid-β deposition in late-life. J. Alzheimer’s Dis..

[45] Jack CR, Knopman DS, Jagust WJ, Shaw LM, Aisen PS, Weiner MW, Petersen RC, Trojanowski JQ (2010). Hypothetical model of dynamic biomarkers of the Alzheimer’s pathological cascade. Lancet Neurol..

[46] Insel PS, Ossenkoppele R, Gessert D, Jagust W, Landau S, Hansson O, Weiner MW, Mattsson N (2017). Alzheimer's Disease Neuroimaging Initiative. Time to amyloid positivity and preclinical changes in brain metabolism, atrophy, and cognition: evidence for emerging amyloid pathology in Alzheimer’s disease. Front. Neurosci..

[47] Insel PS, Mattsson N, Mackin RS, Schöll M, Nosheny RL, Tosun D, Donohue MC, Aisen PS, Jagust WJ, Weiner MW (2016). Alzheimer's Disease Neuroimaging Initiative. Accelerating rates of cognitive decline and imaging markers associated with β-amyloid pathology. Neurology.

[48] Ebert MS, Sharp PA (2010). Emerging roles for natural microRNA sponges. Curr. Biol..

[49] Singh D, Singh PK, Chaudhary S, Mehla K, Kumar S (2012). Exome sequencing and advances in crop improvement. Adv. Genet..

[50] Wu F, Yao PJ (2009). Clathrin-mediated endocytosis and Alzheimer’s disease: an update. Ageing Res. Rev..

[51] Alsaqati M, Thomas RS, Kidd EJ (2018). Proteins involved in endocytosis are upregulated by ageing in the normal human brain: Implications for the development of Alzheimer’s disease. J. Gerontol. Ser. A.

[52] He F, Balling R (2013). The role of regulatory T cells in neurodegenerative diseases. Wiley Interdiscip. Rev. Syst. Biol. Med..

[53] Ruano D, Abecasis GR, Glaser B, Lips ES, Cornelisse LN, de Jong AP, Evans DM, Smith GD, Timpson NJ, Smit AB, Heutink P (2010). Functional gene group analysis reveals a role of synaptic heterotrimeric G proteins in cognitive ability. Am. J. Human Genet..

[54] Pavlowsky A, Chelly J, Billuart P (2012). Emerging major synaptic signaling pathways involved in intellectual disability. Mol. Psychiatry.

[55] Taneja P, Ogier M, Brooks-Harris G, Schmid DA, Katz DM, Nelson SB (2009). Pathophysiology of locus ceruleus neurons in a mouse model of Rett syndrome. J. Neurosci..

