# Supplementary material for: Genetic effects on longitudinal cognitive decline during the early stages of Alzheimer’s disease
Source: Sci Rep. 2021 Oct 6;11:19853. doi: 10.1038/s41598-021-99310-z (PMC8494841; doi:10.1038/s41598-021-99310-z)
Supplement: Supplementary file 1 — Supplementary Information 1. [file 41598_2021_99310_MOESM1_ESM.pdf]

Supplementary Material

Genetic effects on longitudinal cognitive decline during the early stages of Alzheimer's disease

Atul Kumar, PhD[1*]; Maryam Shoai, PhD[2]; Sebastian Palmqvist, MD, PhD[1, 3]; Erik Stomrud, MD, PhD[1, 3]; John Hardy, PhD[2, 4]; Niklas Mattsson-Carlgren, MD, PhD[1, 5, 6, #]; Oskar Hansson, MD, PhD[1, 3, #]

[1]Clinical Memory Research Unit, Department of Clinical Sciences, Lund University, Malmö-20502, Sweden

[2]UK Dementia Research Institute and Department of Neurodegenerative Disease and Reta Lila Weston Institute, UCL Queen Square Institute of Neurology and UCL Movement Disorders Centre, University College London, London, WC1V 6LJ, UK

[3]Memory Clinic, Skåne University Hospital, Malmö, Sweden

[4]Institute for Advanced Study, The Hong Kong University of Science and Technology, Hong Kong SAR, China

[5]Department of Neurology, Skåne University Hospital, Lund, Sweden

[6]Wallenberg Centre for Molecular Medicine, Lund University, Lund, Sweden

# Shared Senior Authors

Email Addresses

Atul Kumar:	atul.kumar@med.lu.se
Maryam Shoai:	m.shoai@ucl.ac.uk
Sebastian Palmqvist:	sebastian.palmqvist@med.lu.se
Erik Stomrud:	erik.stomrud@med.lu.se
John Hardy:	j.hardy@ucl.ac.uk

Niklas Mattsson-Carlgren: niklas.mattsson-carlgren@med.lu.se

Oskar Hansson: oskar.hansson@med.lu.se

*Corresponding Author:

Atul Kumar

Faculty of Medicine

Room No. C1012a

Biomedical Center

Sölvegatan 19

Lund University

Lund-22362Sweden

Mobile: +46762001854

Email: atul.kumar@med.lu.se

1 Method

1.1 Study participants

The study population consisted of 381 cognitively unimpaired (CU) elderly participants and 177 patients with mild cognitive impairment (MCI) from the prospective and longitudinal Swedish BioFINDER sample (clinical trial no. NCT01208675; www.biofinder.se), for which baseline cognitive tests, age, education, gender, Aβ status and at least 3 follow up data for MMSE (including baseline) were available. Among consecutively included patients with mild cognitive symptoms, some were classified as MCI and some as subjective cognitive decline (SCD). Following research guidelines, control participants and patients with SCDs were combined to make CU individuals [1]. The participants were recruited between September 2010 and December 2014 at three different memory clinics as previously described [2, 3]. Briefly, clinical assignment of MCI and SCD was performed after patient recruitment based on a neuropsychological battery as previously described [4]. The subjects were thoroughly assessed for their cognitive complaints by physicians with a special interest in dementia disorders. The inclusion criteria for patients with SCD and MCI were as follows: (i) cognitive complaint; (ii) not fulfilling the criteria for dementia; (iii) a Mini-Mental State Examination (MMSE) score of 24–30 points; (iv) age 60–80 years; and (v) fluent in Swedish. The exclusion criteria were as follows: (i) cognitive impairment that without doubt could be explained by a condition other than prodromal dementias; (ii) severe somatic disease; and (iii) refusing lumbar puncture or neuropsychological investigation. Cognitively normal controls were eligible for inclusion if they (i) were aged 60 years old, (ii) scored 28–30 points on the

Mini-Mental State Examination (MMSE) at the screening visit, (iii) did not have cognitive symptoms as evaluated by a physician, (iv) were fluent in Swedish, (v) did not fulfill the criteria of MCI or any dementia. The exclusion criteria were as follows: (i) presence of significant neurologic or psychiatric disease (e.g., stroke, Parkinson's disease, multiple sclerosis, major depression), (ii) significant systemic illness making it difficult to participate, (iii) refusing lumbar puncture and (iv) significant alcohol abuse. The Regional Ethics Committee in Lund, Sweden, approved the study. All subjects gave their written informed consent.

1.2 Genotyping and preparation of genetic data

Genotyping was conducted using the Illumina platform GSA-MDA v2. Before imputation, subject-level quality control (QC) included removing sexual incompatibility between chip-inferred sex and self-reported sex, low call rates (1% cut-off), and extreme heterozygosity. Relatedness among the samples was eliminated by removing one participant from each pair of close relatives (first or second degree) identified as, $\hat{\pi} \geq 0.0625$. Using PLINK2 [5], multi-dimensional scaling was done to create principal components in genetic analyses to account for ancestry.

Standard QC steps were performed for SNP-level filter to ensure conformity with the reference panel used for imputation (strand continuity, names of the alleles, position and assignments for Ref / Alt). To sum up, for imputation, 685494 high-quality variants (autosomal, non-monomorphic, bi-allelic variants with Hardy–Weinberg Equilibrium (HWE) $P > 5 \times 10^{-8}$ and with a call rate of > 99%) were used.

Imputation was carried out using the Sanger Imputation Server (https://imputation.sanger.ac.uk/) with SHAPEIT for phasing [6], Positional Burrows-Wheeler Transform (PWBT) [7] for imputation and the entire Haplotype Reference Consortium (release 1.1) reference panel [8].

Multi-allelic variants and SNPs with a data imputation score < 0.8 have been excluded as part of post-imputation QC and genotype calls with a posterior likelihood < 0.9 have been set to fail (i.e., hard-called). SNPs with a genotyping rate >0.9 were retained. SNPs with Minor Allele Frequency (MAF) ≥ 5% were taken for the analysis. Further information on the imputation and QC process is detailed in https://rpubs.com/maffleur/452627.

1.3 Statistical Analyses (GWAS)

For the GWAS, we used generalized linear regression models using PLINK2 [5] to test for genetic association with longitudinal MMSE scores. The models were adjusted for age, gender, education, baseline MMSE (not for the intercept), *APOE* ε2 and ε4 count, and top 10 principal components (PC) from the principal component analysis (PCA) on the entire set of genotype data. All the statistical analysis was conducted in R programming (version 4.0.2) using standard R packages.

2 Results

2.1 GWAS of the rate of cognitive decline

We next turned to exploratory analysis of individual genetic variants and rate of cognitive decline adjusting with and without *APOE* ε4 burdens. The genomic inflation factor for association analysis in both analyses was

close to unity ($\lambda = 1$), indicating a subtle population structure. No variant reached genome-wide significance, but 18 variants in *APOE* ε4 burdens adjusted analysis and 22 variants in *APOE* ε4 burdens not adjusted analysis were significant at the suggestive level significance of $p \leq 5 * 10^{-5}$ (supplementary table S29, S30). For both the analysis variant rs10492328 (MAF = 0.27) and rs4747634 (MAF = 0.06) were the top two hits with almost similar level of significance (p = 4.4e-07 and 1.1e-06 respectively). rs10492328 is located near a pseudogene *GLULP5* and variant rs4747634 is located near an RNA gene C10orf16. rs10492328 was associated with cognitive decline (beta = -0.28), whereas rs4747634 showed a protective effect against cognitive decline (0.54). Recent studies have shown that although pseudogenes are not transcribed themselves, they may contribute to the regulation of gene expression [12], making it possible that the variants identified here modulate cognitive decline through regulation of other (unknown) genes

2.2 Education as a proxy for cognitive reserve

Though there was no significant association between PGS-Edu and the cognitive scores, we did find the expected association between education in itself and cognitive decline (supplementary table S7 and S8). To validate PGS-Edu, we performed this supplemental analysis by taking baseline education as a proxy for cognitive reserve. We found the expected results that all education PGSs (PGS-Edu 1-7) we strongly associated with education at baseline (p-value in range of 2.7e-12 to 9.8e-04, after Bonferroni correction). A similar result was observed for all the intelligence PGSs that showed

significant association with the baseline education (p-value in range of 1.4e-04 to 3.7e-02, after Bonferroni correction). However, we did not find any significant association between AD PRSs (PRS-Alz) and education at baseline (supplementary table S39 and supplementary figure 6).

Reference

[1] Jack Jr CR, Bennett DA, Blennow K, Carrillo MC, Dunn B, Haeberlein SB, Holtzman DM, Jagust W, Jessen F, Karlawish J, Liu E. NIA-AA research framework: toward a biological definition of Alzheimer's disease. Alzheimer's & Dementia. 2018 Apr;14(4):535-62.

[2] Mattsson N, Insel PS, Palmqvist S, Stomrud E, Van Westen D, Minthon L, Zetterberg H, Blennow K, Hansson O. Increased amyloidogenic APP processing in APOE ε4-negative individuals with cerebral β-amyloidosis. Nature communications. 2016 Mar 7;7(1):1-7.

[3] Janelidze S, Stomrud E, Palmqvist S, Zetterberg H, Van Westen D, Jeromin A, Song L, Hanlon D, Hehir CA, Baker D, Blennow K. Plasma β-amyloid in Alzheimer's disease and vascular disease. Scientific reports. 2016 May 31;6(1):1-1.

[4] Petrazzuoli F, Vestberg S, Midlöv P, Thulesius H, Stomrud E, Palmqvist S. Brief Cognitive Tests Used in Primary Care Cannot Accurately Differentiate Mild Cognitive Impairment from Subjective Cognitive Decline. Journal of Alzheimer's Disease. 2020 May 11(Preprint):1-1.

[5] Chang CC, Chow CC, Tellier LC, Vattikuti S, Purcell SM, Lee JJ. Second-generation PLINK: rising to the challenge of larger and richer datasets. Gigascience. 2015 Dec 1;4(1):s13742-015.

[6] Delaneau O, Marchini J, Zagury JF. A linear complexity phasing method for thousands of genomes. Nature methods. 2012 Feb;9(2):179-81.
[7] Durbin R. Efficient haplotype matching and storage using the positional Burrows–Wheeler transform (PBWT). Bioinformatics. 2014 May 1;30(9):1266-72.
[8] McCarthy S, Das S, Kretzschmar W, Delaneau O, Wood AR, Teumer A, Kang HM, Fuchsberger C, Danecek P, Sharp K, Luo Y. A reference panel of 64,976 haplotypes for genotype imputation. Nature genetics. 2016 Oct;48(10):1279-83.
[12] Singh RK, Singh D, Yadava A, Srivastava AK. Molecular fossils "pseudogenes" as functional signature in biological system. Genes & Genomics. 2020 Apr 10:1-2.

Supplementary tables

Table S1: PRS-Alz association with the slope of longitudinal MMSE score when not adjusting for Aβ-status

Table S2: PRS-Alz association with the intercept of longitudinal MMSE score when not adjusting for Aβ-status

Table S3: PGS-Int association with the slope of longitudinal MMSE score when not adjusting for Aβ-status

Table S4: PGS-Int association with the intercept of longitudinal MMSE score when not adjusting for Aβ-status

Table S5: PGS-Edu association with the slope of longitudinal MMSE score when not adjusting for Aβ-status

Table S6: PGS-Edu association with the intercept of longitudinal MMSE score when not adjusting for Aβ-status

Table S7: Association of covariates (age, gender and education) with the slope of MMSE for PRS-Alz, PGS-Int and PGS-Edu

Table S8: Association of covariates (age, gender and education) with the Intercept of MMSE for PRS-Alz, PGS-Int and PGS-Edu

Table S9: PRS-Alz and PGS-Int interaction association with the slope of longitudinal MMSE score when not adjusting for Aβ-status

Table S10: PRS-Alz association with the slope of longitudinal MMSE score after adjusting for Aβ-status

Table S11: PRS-Alz association with the intercept of longitudinal MMSE score after adjusting for Aβ-status

Table S12: PGS-Int association with the slope of longitudinal MMSE score after adjusting for Aβ-status

Table S13: PGS-Int association with the intercept of longitudinal MMSE score after adjusting for Aβ-status

Table S14: PRS-Alz association with the slope of longitudinal MMSE score including the Aβ-status-PRS interaction

Table S15: PRS-Alz association with the intercept of longitudinal MMSE score including the Aβ-status-PRS interaction

Table S16: PGS-Int association with the slope of longitudinal MMSE score including the Aβ-status-PRS interaction

Table S17: PGS-Int association with the intercept of longitudinal MMSE score including the Aβ-status-PRS interaction

Table S18: PGS-Edu association with the slope of longitudinal MMSE score including the Aβ-status-PRS interaction

Table S19: PGS-Edu association with the intercept of longitudinal MMSE score including the Aβ-status-PRS interaction

Table S20: Causal Mediation Analysis PRS-Alz 5 using Nonparametric Bootstrap Confidence Intervals with the Percentile Method

Table S21: Association of reduced PRSs with the slope of longitudinal MMSE to test for the change in a p-value of association between reduced PRS and Aβ

Table S22: Association of PRSs consisting of ranked variants with the slope of longitudinal MMSE to identify the independent variant set

Table S23: Association of PRSs consisting of ranked variants (reverse order) with the slope of longitudinal MMSE to identify the dependent variant set

Table S24: Individual-level association of 33 SNPs of PRS-Alz 5 with dichotomized Aβ status

Table S25: Mapping of SNP Variants to Gene for PRS-Alz 5

Table S26: Gene Ontology Enrichment for PRS-Alz 5

Table S27: Reactome Pathway enrichment for PRS-Alz 5

Table S28: Mapping of SNP Variants to Gene for PGS-Int 7

Table S29: Gene Ontology Enrichment for PGS-Int 7

Table S30: Reactome Pathway enrichment for PGS-Int 5

Table S31: PRS-Alz association with the slope of different cognitive measures as an outcome

Table S32: PRS-Alz association with the intercept of different cognitive measures as an outcome

Table S33: PGS-Int association with the slope of different cognitive measures as an outcome

Table S34: PGS-Int association with the intercept of different cognitive measures as an outcome

Table S35: PGS-Edu association with the slope of different cognitive measures as an outcome

Table S36: PGS-Edu association with the intercept of different cognitive measures as an outcome

Table S37: Association Results for rate of cognitive decline based on MMSE adjusted for APOE ε2 and ε4 count

Table S38: Association Results for rate of cognitive decline based on MMSE not adjusted for APOE ε2 and ε4 count

Table S39: PRS/PGS association with the baseline education as a proxy for cognitive reserve

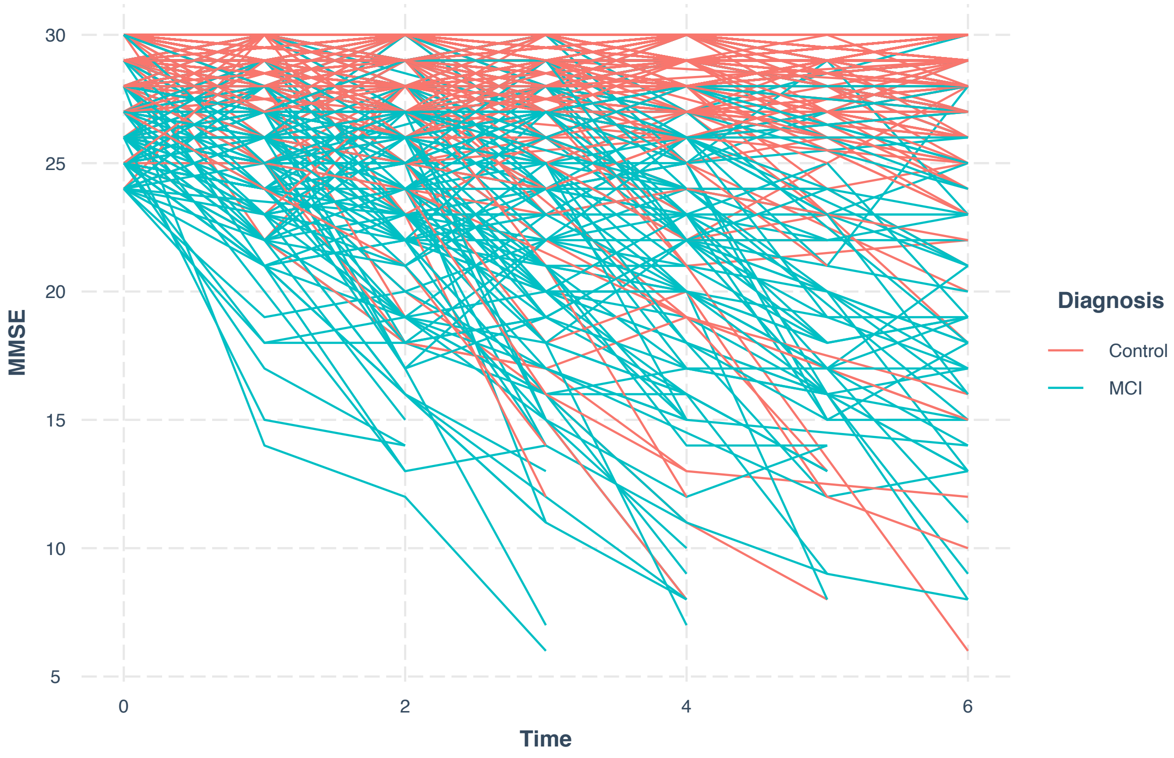

Figure S1: Spaghetti plot showing the individual trajectories on the cognitive performance based on Mini-Mental State Examination (MMSE) score. The X-axis shows the follow-up time in years and the y-axis shows the MMSE score

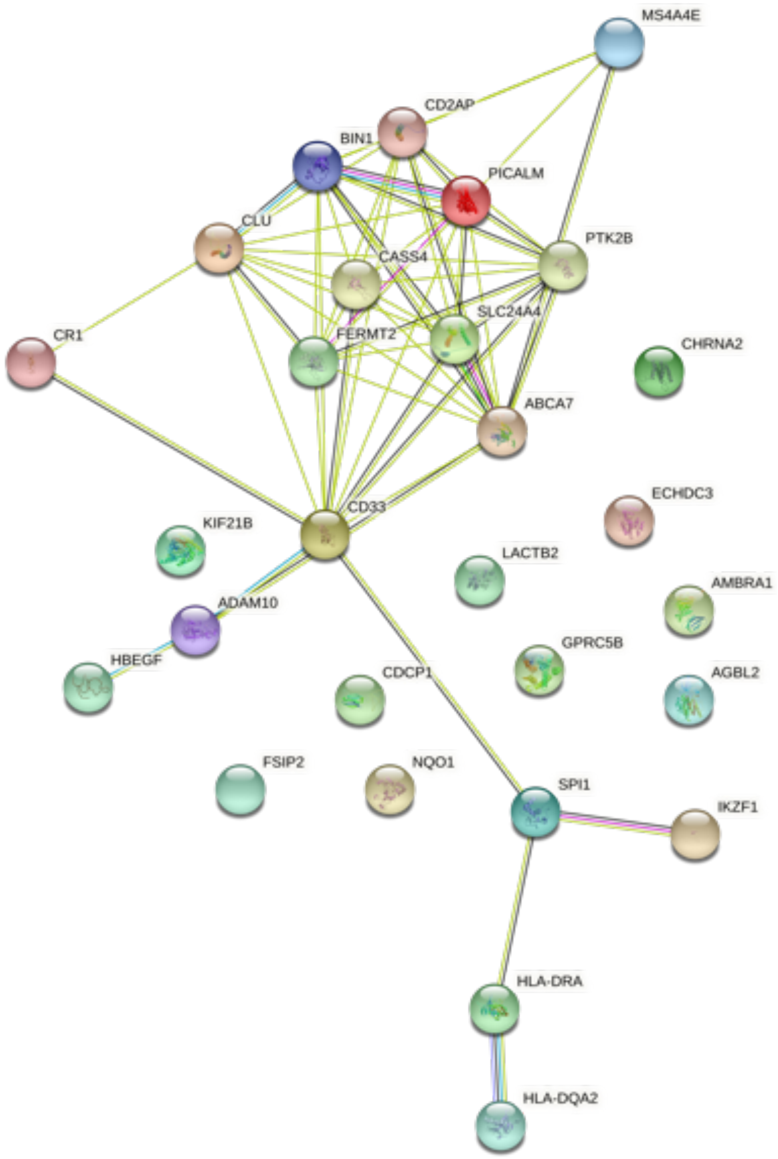

Figure S2: Protein-Protein Interaction network for the protein-coding genes of the SNPs of Alz PRS 8. Nodes represent the proteins and edges indicate both functional and physical protein association. The edge line thickness indicates the strength of data support at an interaction score ≥ 0.4.

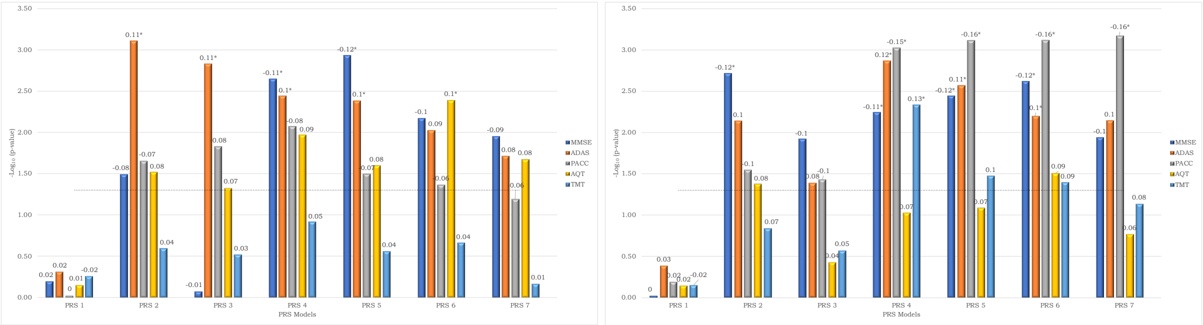

a b

Figure S3: Associations of Polygenic Scores of AD with four other cognitive measures along with MMSE: ADAS-Cog delayed recall test (A test of episodic memory from Alzheimer's Disease Assessment Scale), AQT (A Quick Test: A test of processing speed and executive function), TMT-B (Trail Making Test part B: A test of processing speed and executive function) and PACC (Preclinical Alzheimer Cognitive Composite: A composite sensitive to very early cognitive decline in AD). a) PRS-Alz association with the slope of cognitive measure. b) PRS-Alz association with the intercept of cognitive measure. The x-axis represents the 7 different PRS models at different p-value thresholds based on the GWAS summary statistics (PRS1 ≤ 0.05, PRS2 ≤ 5e-3, PRS3 ≤ 5e-4, PRS4 ≤ 5e-5, PRS5 ≤ 5e-6, PRS6 ≤ 5e-7, PRS7 ≤ 5e-8). The models were adjusted for age, gender, education, baseline cognitive measure score (not for the intercept), *APOE* ε2 and ε4 count and the top 10 principal components (PC) from the principal component analysis (PCA) on the entire set of genotype data. The y-axis shows the negative log of the p-value for the significance of associations between PRS models with slope and intercept of longitudinal cognitive measure score. The values on the top of each bar show the effect size (beta-coefficient) of the association. The horizontal dotted line shows the p-value

threshold of 0.05. *These PRSs were significant after Bonferroni-correction at p-value < 0.05.

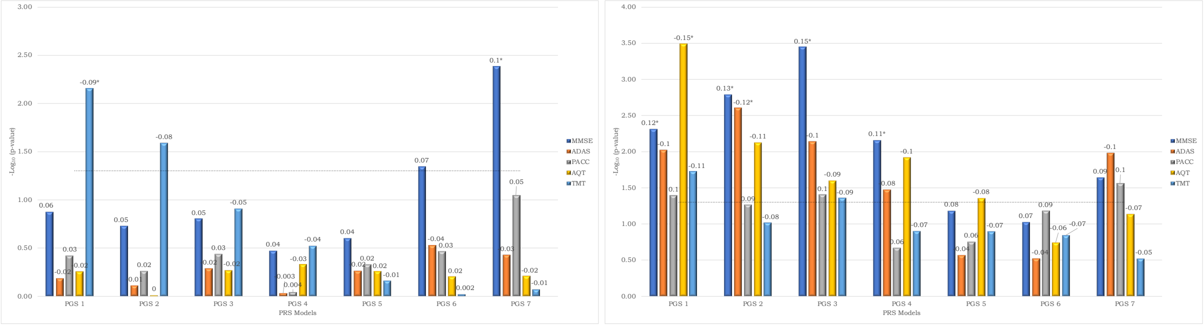

a b

Figure S4: Associations of Polygenic Scores of intelligence with four other cognitive measures along with MMSE: ADAS-Cog delayed recall test (A test of episodic memory from Alzheimer's Disease Assessment Scale), AQT (A Quick Test: A test of processing speed and executive function), TMT-B (Trail Making Test part B: A test of processing speed and executive function) and PACC (Preclinical Alzheimer Cognitive Composite: A composite sensitive to very early cognitive decline in AD). a) PGS-Int association with the slope of cognitive measure. b) PGS-Int association with the intercept of cognitive measure. The x-axis represents the 7 different PGS models at different p-value thresholds based on the GWAS summary statistics (PGS1 ≤ 0.05, PGS2 ≤ 5e-3, PGS3 ≤ 5e-4, PGS4 ≤ 5e-5, PGS5 ≤ 5e-6, PGS6 ≤ 5e-7, PGS7 ≤ 5e-8). The models were adjusted for age, gender, education, baseline cognitive measure score (not for the intercept), *APOE* ε2 and ε4 count and the top 10 principal components (PC) from the principal component analysis (PCA) on the entire set of genotype data. The y-axis shows the negative log of the p-value for the significance of associations

between PGS models with slope and intercept of longitudinal cognitive measure score. The values on the top of each bar show the effect size (beta-coefficient) of the association. The horizontal dotted line shows the p-value threshold of 0.05. *These PGSs were significant after Bonferroni-correction at p-value < 0.05.

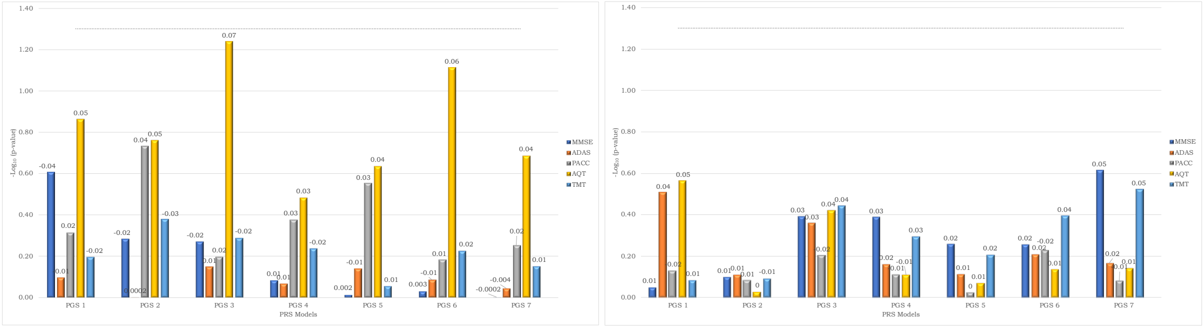

a b

Figure S5: Associations of Polygenic Scores of education with four other cognitive measures along with MMSE: ADAS-Cog delayed recall test (A test of episodic memory from Alzheimer's Disease Assessment Scale), AQT (A Quick Test: A test of processing speed and executive function), TMT-B (Trail Making Test part B: A test of processing speed and executive function) and PACC (Preclinical Alzheimer Cognitive Composite: A composite sensitive to very early cognitive decline in AD). a) PGS-Edu association with the slope of cognitive measure. b) PGS-Edu association with the intercept of cognitive measure. The x-axis represents the 7 different PGS models at different p-value thresholds based on the GWAS summary statistics (PGS1 ≤ 0.05, PGS2 ≤ 5e-3, PGS3 ≤ 5e-4, PGS4 ≤ 5e-5, PGS5 ≤ 5e-6, PGS6 ≤ 5e-7, PGS7 ≤ 5e-8). The models were adjusted for age, gender, education, baseline cognitive measure score (not for the intercept),

APOE ε2 and ε4 count and the top 10 principal components (PC) from the principal component analysis (PCA) on the entire set of genotype data. The y-axis shows the negative log of the p-value for the significance of associations between PGS models with slope and intercept of longitudinal cognitive measure score. The values on the top of each bar show the effect size (beta-coefficient) of the association. The horizontal dotted line shows the p-value threshold of 0.05.

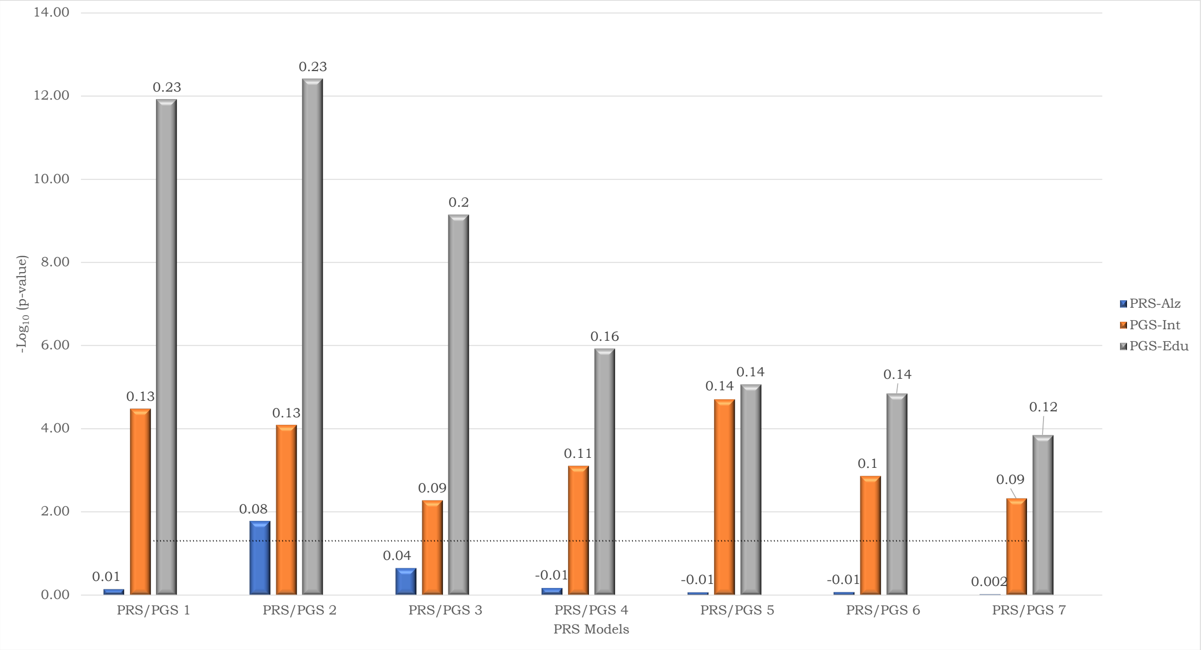

Figure S6: Associations of Polygenic Scores of AD, intelligence and education with education at baseline as a proxy for cognitive reserve. The x-axis represents the 7 different PRS/PGS models at different p-value thresholds based on the GWAS summary statistics (PRS1 ≤ 0.05, PRS2 ≤ 5e-3, PRS3 ≤ 5e-4, PRS4 ≤ 5e-5, PRS5 ≤ 5e-6, PRS6 ≤ 5e-7, PRS7 ≤ 5e-8). The models were adjusted for age, gender, *APOE* ε2 and ε4 count and the top 10 principal components (PC) from the principal component analysis (PCA) on the entire genotype data set. The y-axis shows the negative log of the p-value

for the significance of associations between PRS models with education at baseline. The values on the top of each bar show the effect size (beta-coefficient) of the association. The horizontal dotted line shows the p-value threshold of 0.05. All the Education PGSs (PGS-Edu) and intelligence PGSs (PGS-Int) were significant after Bonferroni-correction at p-value < 0.05.
